# Manganese-dependent iron-superoxide dismutase drives *Acinetobacter baumannii* fitness during oxidative stress

**DOI:** 10.1016/j.jbc.2025.110549

**Published:** 2025-08-05

**Authors:** Ashish Kumar Ray, Somok Bhowmik, Snehlata Saini, Arsalan Hussain, Perwez Bakht, Shivam Pandey, Ranjana Pathania

**Affiliations:** 1 Department of Biosciences and Bioengineering, Indian Institute of Technology, Roorkee, Uttarakhand, India; 2 Centre of Excellence in Disaster Mitigation and Management, Indian Institute of Technology, Roorkee, Uttarakhand, India

**Keywords:** metalloenzyme, metal ion–binding residues, Cu/Zn–SOD, reactive oxygen species, bacterial pathogenesis

## Abstract

Superoxide dismutase (SOD), a widely distributed metalloenzyme across all domains of life, mitigates the harmful effects of reactive oxygen species generated during oxidative stress. The catalytic activity of SOD depends on specific metal cofactor, which is determined by bioavailability, structural compatibility, and environmental factors. The nosocomial pathogen *Acinetobacter baumannii* has been able to thrive under oxidative stress with SODs imparting a major role in curbing this distress. However, the functional role of two encoded SODs, namely SodB and SodC in *A. baumannii*, is poorly understood in mitigating oxidative stress. Furthermore, the metal ion specificities of these SodB and SodC families exemplify a knowledge gap, as the metal ion utilized by individual family members cannot be reliably predicted. Our study unveils the specific metal cofactors utilized by SodB and SodC and their role in quenching host-mediated oxidative stress during infection by *A. baumannii* 5075 (AB5075), a hypervirulent and multidrug-resistant strain. The study reveals that SodB primarily utilizes Mn^2+^, whereas SodC employs Cu^2+^ to achieve optimal catalytic efficiency in superoxide dismutation. The oxidative stress response in AB5075 favors SodB over SodC, highlighting the critical role of Mn-dependent SodB in counteracting oxidative stress. Furthermore, we demonstrated that mutations in metal-binding residues (SodB^H28A^, SodB^D164A-H168A^, SodC^H87A-H89A^, and SodC^H165A^) led to significant impairment of SOD activity, thus highlighting the importance of these residues in catalytic function and preference for metal ion cofactor. The study shows that SodB has identical metal ion–binding residues for both Fe^2+^ and Mn^2+^ but is only active with Mn^2+^ ion.

Metalloproteins are fundamental to numerous biological processes, with metal ions, such as manganese (Mn^2+^), iron (Fe^2+^), zinc (Zn^2+^), and copper (Cu^2+^), serving as essential cofactors for nearly half of all known enzymes (1, 2, 3). Among these enzymes, the superoxide dismutase (SOD) family plays a critical role in cellular defense against oxidative stress by catalyzing the conversion of superoxide radicals (O_2_^•−^) into molecular oxygen (O_2_) and hydrogen peroxide (H_2_O_2_) (4). Thus, SODs play a vital role in maintaining cellular redox balance and to mitigate the harmful effects of reactive oxygen species (ROS) generated during metabolic processes and environmental stress (4). SODs are classified into distinct families based on their preferred metal cofactors, primarily manganese-dependent SOD (Mn-SOD), iron-dependent SOD (Fe-SOD), copper/zinc-dependent SOD (Cu/Zn-SOD), and nickel-dependent SOD (5, 6, 7). In bacteria, Mn-SODs (encoded by the *sodA*) and Fe-SODs (encoded by the *sodB*) are located in the cytoplasm. In contrast, Cu/Zn-SODs (encoded by the *sodC*) are found in the periplasm of many Gram-negative bacteria (8, 9, 10).

The metal ions enhance enzyme activity by aiding in catalysis, stabilizing protein structures, and enabling electron transfer, highlighting their significance in biochemical functions. The metal ion specificity of SODs influences both their catalytic efficiency and adaptability to diverse environmental conditions. Thus, studying these cofactors becomes essential for understanding bacterial pathophysiology. However, the factors of metal ion specificity in SODs remain poorly characterized, complicating our understanding of how bacterial pathogens exploit these enzymes to evade host defenses. During bacterial infection, the host recruits phagocytic cells as a primary defense mechanism to eliminate invading bacteria (11). Phagocytic cells sequester free metal ions at the site of infection, a phenomenon known as “nutritional immunity,” and induce oxidative stress by generating free radicals (12, 13). This disrupts the bacterial oxidative stress response by limiting the activity of bacterial SODs, which depend on specific metal ions as cofactors for their catalytic function (14).

The Gram-negative, multidrug-resistant bacterium *Acinetobacter baumannii* has emerged as a significant nosocomial pathogen (15, 16). The success of *A. baumannii* to evade host immune response exhibits its ability to resist oxidative stress. To thrive in harsh environments, it employs SOD as a primary defense against oxidative stress (17). Whole-genome sequencing of *A. baumannii* American Type Culture Collection (ATCC) 17978 identified two SOD genes, A1S_2343 and A1S_3143, which encode putative Fe/Mn-SOD and Cu/Zn-SOD, respectively. The putative *sodB* has been implicated in mediating motility, pathogenicity, resistance to oxidative stress and antibiotics in *A. baumannii* ATCC 17978 (17). AB5075, a hypervirulent and multidrug-resistant strain, embodies a better representation of the clinically relevant pathogenic potential of *A. baumannii* to withstand oxidative stress (18). However, the specific metal ions required for the catalytic activity of these SODs in *A. baumannii* remain to be elucidated. In this study, we focus on two distinct SODs, designated SodB and SodC, derived from *A. baumannii* strain 5075 (AB5075). Through a combination of *in vivo* and *in vitro* assays, we elucidate the metal specificity and functional significance of these enzymes in the pathogenesis of *A. baumannii*. Our findings reveal that SodB in AB5075 exclusively utilizes Mn^2+^ as its cofactor instead of Fe^2+^, whereas SodC relies on Cu^2+^ rather than Zn^2+^. Also, we show that AB5075 preferentially relies on SodB over SodC, enabling it to more effectively combat host-induced oxidative stress. Furthermore, we investigate the impact of targeted mutations in key metal ion–binding residues, showing that these alterations significantly impair SOD activity, disrupting redox functionality of metal cofactors. This study provides insights into the metal cofactors utilized by AB5075 SODs, thereby enhancing our understanding of the mechanisms employed by bacterial pathogens to thrive in hostile environments.

## Results

### SodB plays a critical role as compared with SodC in AB5075 to resist oxidative stress

**Figure 1:**
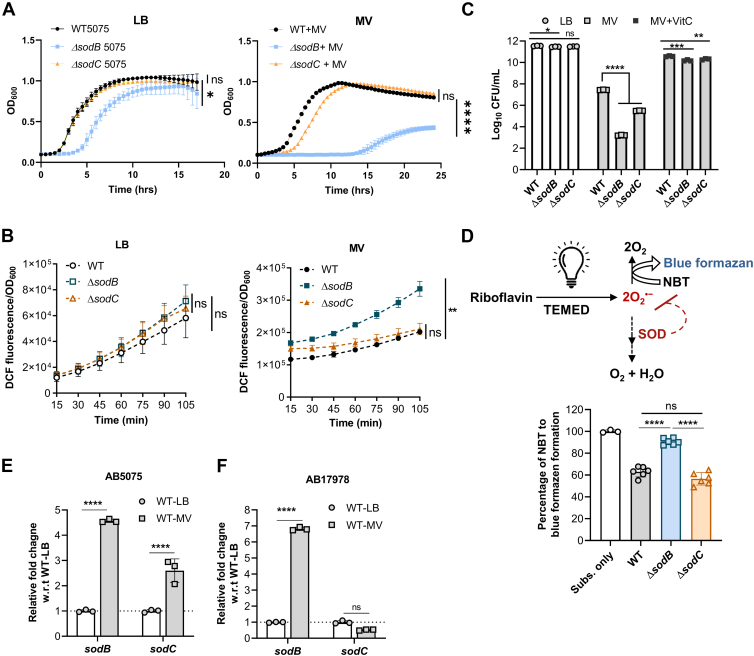
**SodB plays a critical role as compared with SodC in AB5075 to resist oxidative stress.***A,* AB5075 (WT), *ΔsodB*, and *ΔsodC* strains were grown in LB medium supplemented with or without 150 μM methyl viologen (MV). The data represent the mean of four biological replicates ± SD. *B,* bacterial intracellular ROS generation in the presence or the absence of MV was determined by measuring the fluorescence of DCF dye. The data represent the mean of four biological replicates ± SD after an absorbance at 600 nm normalization. *C,* WT, *ΔsodB*, and *ΔsodC* strains were grown to an absorbance of 0.6 at 600 nm in LB medium and pulsed with MV (250 μM) for 120 min. Vitamin C (VitC) at a concentration of 0.5 mg/ml was supplemented to restore the growth defect under MV-induced oxidative stress. The data represent the mean of recovered CFU of four biological replicates, each in technical triplicates ± SD. *D,* total SOD activity was determined using nitroblue tetrazolium (NBT) as a substrate. The photochemical reduction of NBT to blue formazan complex in the presence of riboflavin can be determined at an absorbance at 560 nm (*upper panel*). An equal amount of bacterial cell lysates, grown in LB-medium supplemented with MV, were used in this assay. Active SOD causes an inhibition of the photochemical reduction of NBT to *blue* formazan complex (*lower panel*). The data represent the mean of six biological replicates ± SD except for only substrate. Statistical significance was determined using a multiple comparison one-way ANOVA test with the Sidak correction for multiple comparisons comparing the means of each group to one another. *E* and *F,* the transcript levels of sodB and sodC were determined in WT-5075 or WT-17978 cells grown in LB medium to an absorbance of ∼0.6 at 600 nm and pulsed with MV (250 μM) for 120 min by quantitative RT–PCR. The data represent the mean ± SD. Statistical significance was determined using a multiple comparison two-way ANOVA test with the Sidak correction for multiple comparisons comparing the means of each group to one another. ∗ denotes *p* < 0.05, ∗∗ denotes *p* < 0.01, ∗∗∗ denotes *p* < 0.001, ∗∗∗∗ denotes *p* < 0.0001, ns denotes not significant. CFU, colony-forming unit; DCF, dichlorofluorescein; LB, lysogeny broth; ROS, reactive oxygen species; SOD, superoxide dismutase.

The nosocomial pathogen *A. baumannii* demonstrates a remarkable ability to withstand both endogenous and exogenous ROS through the antioxidant activity of SOD. To investigate the distinct roles of SodB and SodC in the oxidative stress response of AB5075, transposon mutants of both SodB (Δ*sodB*) and SodC (Δ*sodC*) were utilized. The growth of WT, Δ*sodB*, and Δ*sodC* strains was assessed in lysogeny broth (LB broth), with or without methyl viologen (MV), a compound known to induce oxidative stress by generating ROS, specifically superoxide radicals (19, 20). In the absence of MV, Δ*sodC* mutant displayed similar growth patterns comparable to the WT, whereas the Δ*sodB* mutant showed an extended lag phase and a minimal growth defect (Fig. 1*A*). However, MV supplementation in LB led to a growth delay in the Δ*sodC* mutant, characterized by an extended lag phase, whereas the Δ*sodB* mutant showed a significant growth defect compared with WT (Fig. 1*A*). This study highlights the essential function of SODs in managing ROS toxicity and demonstrated that SodB plays a significant role than SodC in protecting AB5075 against MV-induced oxidative stress.

To assess intracellular ROS production, we measured the ROS levels in WT, *ΔsodB*, and *ΔsodC* mutants following incubation with MV. ROS generation was detected using 2′,7′-dichlorofluorescein dye, a fluorescent dye commonly employed as an indicator of ROS accumulation in cells. Notably, *ΔsodB* mutant exhibited a significant, time-dependent increase in ROS levels as compared with both WT and *ΔsodC* mutant upon MV treatment (Fig. 1*B*). This observation highlighted that the presence of SodB is important in curbing the ROS, particularly in the breakdown of intracellular ROS. Further validation was conducted by using vitamin C (VitC) as a free radical quencher (21, 22). The survival of both *ΔsodB* and *ΔsodC* mutants was reduced in LB medium supplemented with MV in the absence of VitC. However, their growth defects were restored with the addition of VitC (Fig. 1*C*). This result suggested that the growth defects observed in the mutants under oxidative stress can be rescued by antioxidants, further supporting the critical role of SodB and SodC in mitigating ROS-induced cellular damage.

To further evaluate SOD activity, an NBT (nitroblue tetrazolium) assay was performed on WT, *ΔsodB*, and *ΔsodC* cells exposed to MV. This assay measures the formation of a blue formazan complex, which reflects the presence of free radicals and is inhibited by active SODs. The results showed similar SOD activity in WT and *ΔsodC* cells, whereas the *ΔsodB* mutant displayed significantly elevated blue formazan formation, indicative of diminished SOD activity (Fig. 1*D*). In addition, to assess the expression of SOD genes in oxidative stress conditions, the transcript levels of *sodB* and *sodC* in WT cells under MV-induced oxidative stress were quantified using quantitative RT–PCR. A significant ∼5-fold upregulation of *sodB* transcripts was observed compared with ∼3-fold upregulation of *sodC* under oxidative stress condition (Fig. 1*E*). Since both SODs of AB5075 share identical sequences with their homologs in *A. baumannii* ATCC 17978, we also measured their transcriptional abundance in WT-17978 cells under similar oxidative stress condition. In agreement with the findings in AB5075, *sodB* transcripts were significantly upregulated compared with *sodC* (Fig. 1*F*). Collectively, these results underscore the critical role of SodB in mitigating oxidative stress in AB5075, highlighting its importance over SodC in managing superoxide-induced toxicity.

### SodB contributes to AB5075 fitness effectively as compared with SodC by reducing host-induced oxidative stress during infection

**Figure 2:**
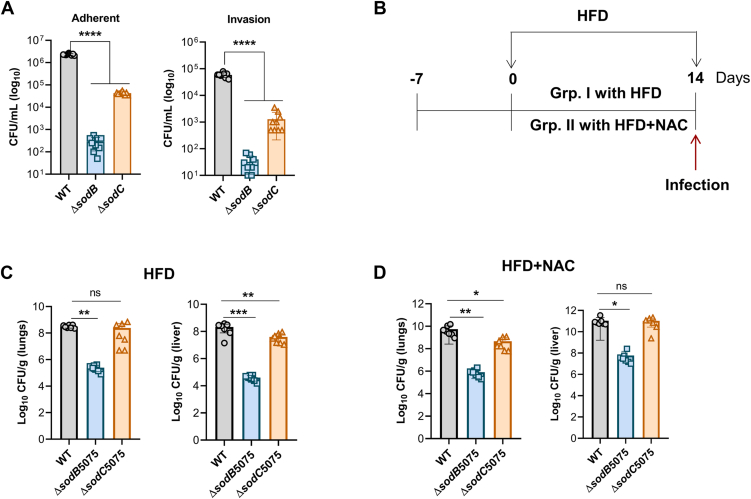
**SodB contributes to AB5075 fitness effectively as compared with SodC by reducing host-induced oxidative stress during infection.***A,* the human alveolar basal epithelial cells (A549) were infected with WT, ΔsodB, or ΔsodC strains at a multiplicity of infection of 100 for 4 h. Adhesion efficiency was determined by counting the number of bacteria per well after washing away nonadherent cells and treating with 0.1% Triton X-100 (*left panel*). Invasion efficiency was measured as the number of bacteria per well using the gentamicin protection assay (*right panel*). The data represent the mean of nine biological replicates ± SD. Statistical significance was determined using a multiple comparison one-way ANOVA test with the Sidak correction for multiple comparisons comparing the means of each group to one another. *B,* a schematic representation of the mouse experiment utilizing a high-fat diet (HFD) with or without *N*-acetylcysteine (NAC) supplementation in the drinking water. *C* and *D,* enumeration of the bacterial burden recovered from the lungs and liver of mice (n = 7) infected with WT, ΔsodB, or ΔsodC strains at 36 h postinfection (hpi). Data are presented as mean ± SD. Statistical significance was determined using the one-way ANOVA test with Tukey's multiple comparisons. ∗ denotes *p* < 0.05, ∗∗ denotes *p* <0.01, ∗∗∗ denotes *p* < 0.001, ∗∗∗∗ denotes *p* < 0.0001, ns denotes not significant.

The prominent role of SodB as compared with SodC in AB5075 oxidative stress resistance emphasizes its significance in regulating bacterial survival and fitness within the host's oxidative environment. During epithelial cell invasion, bacteria encounter cell-mediated oxidative stress, which imposes a significant challenge to their survival and virulence. The overexpression of Cu/Zn-SOD in *Escherichia coli* has been shown to confer a selective advantage by neutralizing ROS, thereby enhancing bacterial fitness under such conditions (23). To investigate the roles of SodB and SodC in epithelial cell's adhesion and invasion, bacterial infection assays were conducted using A549 epithelial cells with *ΔsodB* and Δ*sodC* mutants of AB5075. Both mutants exhibited significant defects in their ability to adhere to and invade epithelial cells compared with the WT strain (Fig. 2*A*). Notably, the Δ*sodB* mutant displayed a greater reduction in adhesion and invasion capabilities compared with the Δ*sodC* mutant (Fig. 2*A*). This implies that SodB profoundly enabled the bacterial cells to counter oxidative stress and establish interactions with host cells. The observed adhesion defects in both mutants were directly correlated with their invasion deficiencies, indicating that efficient adhesion to host epithelial cells is a prerequisite for successful invasion. To establish successful bacterial infection, SodB and to a lesser extent, SodC facilitated the bacterial adhesion and invasion during oxidative stress encountered within the host environment. In addition to protecting against oxidative stress, SOD activity has been implicated in the regulation of genes involved in exopolysaccharide production and biofilm formation on abiotic surfaces, as reported in *Klebsiella pneumoniae* (24). In this study, we observed that both Δ*sodB* and Δ*sodC* mutants of AB5075 exhibited significant defects in biofilm formation compared with the WT strain (Fig. S2*A*) with Δ*sodB* showing greater defect than Δ*sodC*. These findings highlight the critical role of SodB in not only mitigating oxidative stress during infection but also in regulating other essential physiological processes, such as biofilm formation, which are crucial for bacterial adaptation and survival in various abiotic hostile environments.

ROS are inevitable metabolic byproducts in aerobic organisms, including pathogenic microbes. This oxidative burden is exacerbated during host immune responses, particularly because of the oxidative burst generated to combat infections. Under a high-fat diet (HFD), which disrupts redox homeostasis and enhances oxidative stress (25), pathogenic bacteria must deploy antioxidant mechanisms to ensure survival within the host environment (26). A preliminary investigation was conducted to evaluate ROS generation in HFD-fed C57BL/6J male mice preceding infection studies. Utilizing the Mouse Reactive Oxygen Species Modulator 1 (ROMO1) ROS ELISA kit, a comparative analysis of lung and liver tissues demonstrated significantly elevated ROS levels in HFD-fed mice relative to normal diet controls, thereby confirming diet-induced oxidative stress in these tissues (Fig. S2, *B* and *C*). To evaluate the effects of HFD-induced oxidative stress on AB5075 survival during systemic infection, C57BL/6J male mice were fed with an HFD for 2 weeks prior to being infected with WT, Δ*sodB*, and Δ*sodC* strains (Fig. 2*B*). After 36 h, bacterial burden was assessed in the lungs and liver of the infected mice. The Δ*sodB* mutant exhibited a significant reduction in survival in both organs compared with the WT strain (Fig. 2*C*). In contrast, the *ΔsodC* mutant showed survival comparable to the WT in the lungs but displayed a substantial decrease in the liver. These results demonstrated the major role of SodB in enabling AB5075 to combat oxidative stress across multiple organs, while also suggesting a tissue-specific role for SodC in bacterial survival during infection. To further assess the impact of HFD-induced oxidative stress on AB5075 fitness, *N*-acetylcysteine (NAC), a well-characterized antioxidant that scavenges ROS (27, 28), was added to the drinking water of infected mice. NAC treatment significantly increased the bacterial burden in the lungs and liver of WT cells. In contrast, *ΔsodB* and *ΔsodC* mutants exhibited minimal changes in bacterial burden in the lungs but showed a significant increase in the liver. These findings confirm that oxidative stress, associated with an HFD, poses a substantial challenge to bacterial survival (Fig. 2*D*). Intriguingly, in NAC-treated mice, the survival of Δ*sodC* cells in the liver was comparable to that of WT cells, suggesting a compensatory mechanism under reduced oxidative stress conditions (Fig. 2*D*). In contrast, while NAC supplementation improved the fitness of Δ*sodB* cells in the liver, the Δ*sodB* mutant continued to display a significant survival defect compared with WT and Δ*sodC* strains (Fig. 2*D*). These results indicate that SodB and SodC have roles beyond oxidative stress mitigation in liver, likely contributing to other essential processes critical for AB5075 fitness during infection. In addition to neutralizing superoxide radicals, SOD enzymes influence various physiological processes, including metabolic reprogramming, biofilm and capsule synthesis, cell morphology maintenance, and the ability to colonize and persist in nasopharyngeal and chronic lung infections (24). Collectively, these results emphasize the indispensable role of SodB in promoting AB5075 survival during host-induced oxidative stress and reinforce the broader significance of SODs in bacterial pathogenicity.

### SodB in AB5075 reliably utilizes Mn, not Fe, as a cofactor to enhance its catalytic activity

**Figure 3:**
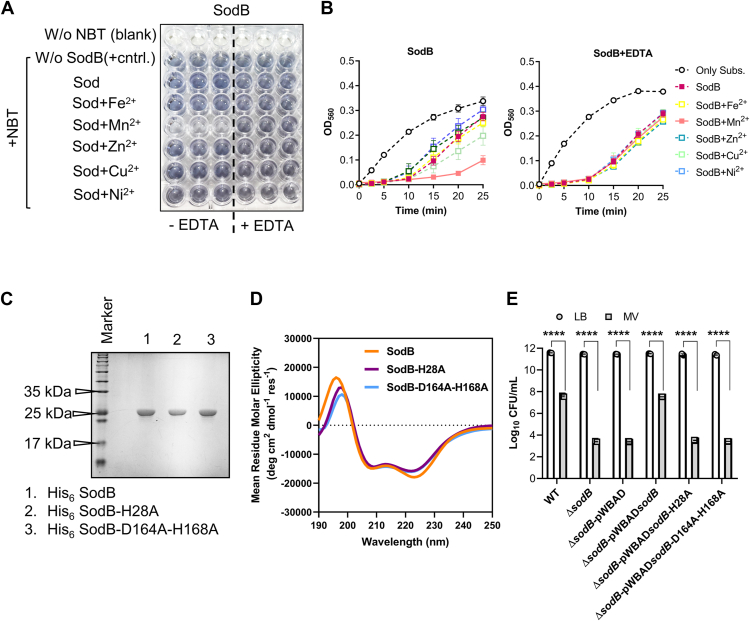
**SodB in AB5075 reliably utilizes manganese (Mn), not iron (Fe), as a cofactor to enhance its catalytic activity.***A,* AB5075 SodB was expressed in *Escherichia coli* BL21 (DE3) strain and FPLC purified followed by treatment with Chelex 100 to remove any bound metal ions. The activity of purified SodB was determined using NBT as a substrate in Hepes buffer. An equal amount of protein (100 nM) and an equimolar ratio of metal ions were used in this assay. Active SOD causes an inhibition of the photochemical reduction of NBT to blue formazan formation, which can be visualized by the naked eye. EDTA was used at a concentration of 50 mM to inhibit the metal-dependent activity of SOD, indicated as +EDTA, whereas the absence of EDTA is denoted as −EDTA. The blank represents the protein in buffer without NBT added to the substrate buffer, whereas wells containing substrate buffer with NBT serve as a positive control without protein (positive control). The *dotted box* indicates the Mn-dependent efficient activity of SodB (image was captured after 1 h of assay). *B,* SodB protein (100 nM) was incubated with the specified metal ions, with or without EDTA, and the formation of *blue* formazan was measured at an absorbance at 560 nm at the designated time points. Reactions containing substrate buffer with NBT serve as a positive control without protein (only subs.). *C,* SDS-PAGE analysis displaying the sizes of His-tagged native SodB, SodB-H28A, and SodB-D164A–H168A. *D,* overlaid CD spectra (n = 1) of native SodB, SodB-H28A, and SodB-D164A–H168A, indicating similar secondary structure content among the three proteins. *E,* WT, ΔsodB, and ΔsodB complemented strains were grown to an absorbance of ∼0.6 at 600 nm in LB medium and pulsed with MV (250 μM) for 120 min. The data represent the mean of recovered CFU of three biological replicates, each in technical triplicates ± SD. Statistical significance was determined using a multiple comparison one-way ANOVA test with the Sidak correction for multiple comparisons comparing the means of each group to one another. ∗∗∗∗ denotes *p* < 0.0001. CFU, colony-forming unit; LB, lysogeny broth; MV, methyl viologen; NBT, nitroblue tetrazolium; SOD, superoxide dismutase.

SODs are metalloenzymes that require metal ions as cofactors for their catalytic activity. However, the metal ion specificity of bacterial SODs remains poorly understood, complicating our ability to decipher how bacterial pathogens manipulate host immune responses to facilitate infection. To investigate metal ion preference and catalytic activity of critical antioxidant SodB in AB5075, we performed a comprehensive analysis of its metal ion–dependent function. The crystal structure of SodB from *Acinetobacter* sp. Ver3 revealed key Fe-binding residues, including H28, H80, D164, and H168 (29). Interestingly, some enzymes, such as those in the aerotolerant anaerobe *Porphyromonas gingivalis*, exhibit cambialistic behavior, enabling them to utilize either Mn or Fe ion as cofactors depending on oxygen availability (30). This adaptability in metal ion usage allows these enzymes to adapt to varying environmental conditions, which may also be relevant for understanding the metal ion preference of SodB in AB5075 under different physiological conditions. Multiple sequence alignment of SodB proteins (Fig. S1*A*) revealed a high degree of sequence conservation across both bacterial and eukaryotic species, particularly at residues known to be critical for catalytic function. Key histidine residues involved in metal cofactor coordination, as well as aspartate residues important for maintaining structural and enzymatic integrity, were consistently conserved. This conservation across phylogenetically diverse organisms indicates that the SodB domain performs a fundamental and evolutionarily preserved role in oxidative stress defense (Fig. S1*B*). To further investigate this, we used the Metal Ion–Binding site prediction server (MIB), which employs structural templates and known coordination geometries to identify potential metal-binding sites—where higher scores indicate a greater likelihood of binding (31). In addition, we assessed the binding affinities of Mn^2+^ and Fe^2+^ for SodB. We assessed the binding affinities of Mn^2+^ and Fe^2+^ for SodB, and the results revealed that both Mn and Fe are capable of binding to SodB at identical residues, with binding scores of 5.415 for Mn^2+^ and 6.133 for Fe^2+^ (Table S1), suggesting that SodB can potentially utilize either metal ion as a cofactor. To validate the MIB-predicted metal-binding residues, we employed AlphaFill and Schrödinger to confirm the identified binding sites and visualize the spatial organization of metal ions within the catalytic site. The observed metal coordination environment was consistent with the MIB predictions, reinforcing the accuracy of the identified binding residues. The purified SodB in its native form shows minimal stochiometric ratio of Fe, Mn, Zn, Cu, and Ni, which gets further depleted on chelex treatment. The reintroduction of these metal ions increases the stoichiometric level to a higher fold (Fig. S4). In our *in vitro* assays with purified SodB, we found that the enzyme exhibited catalytic activity only in the presence of Mn^2+^ (Table 1; Fig. 3, *A* and *B*), indicated by color change, highlighting a clear preference for Mn^2+^ as the cofactor. To further investigate this preference, we focused on the predicted Mn^2+^-binding sites within SodB and generated two site-directed mutants: SodB-H28A and SodB-D164A–H168A. The SodB-H28A mutant showed a reduced binding score for Mn^2+^, suggesting a weakened interaction at this residue, whereas the SodB-D164A–H168A mutant showed no detectable binding to Mn^2+^ (Table S1). These results provide strong evidence that specific residues within the binding pocket are crucial for the preferential binding of Mn^2+^ and the catalytic function of SodB in AB5075. This finding redefines annotation of SodB, from referring Fe-SOD to Mn-SOD based on its exclusive catalytic reliance on Mn as the sole cofactor. To assess the enzymatic activity of these mutants, both SodB-H28A and SodB-D164A-H168A mutants were purified, and their sizes and structural characteristics were analyzed using SDS-PAGE and CD spectroscopy, respectively. SDS-PAGE analysis revealed that all the purified proteins had similar sizes (Fig. 3*C*), and CD spectra indicated comparable secondary structures (Fig. 3*D*). However, enzyme activity assays showed that the SodB-H28A mutant had reduced enzymatic activity, whereas the SodB-D164A–H168A mutant did not exhibit any detectable activity (Table 1). When assessed in the presence of Mn, the SodB-H28A mutant showed minimal activity, and the SodB-D164A–H168A mutant displayed no activity (Table 1). The site-directed mutagenesis study in the metal-binding site highlights the critical role of residues at positions 164 and 168 in the catalytic function of SodB. Despite these findings, our attempts to directly assess the binding of Mn to both native and mutant SodB proteins using binding assays did not yield detectable interactions (data not shown). These results suggest that while the residues involved may not directly coordinate with the metal, they are essential for the catalytic activity and maintaining structural integrity.

**Table 1:** SOD activity of purified native SodB and mutant SodB proteins

Enzyme	Enzyme IC_50_ (nM)										
	Only	Fe		Mn		Zn		Cu		Ni	
	-	−EDTA	+EDTA	−EDTA	+EDTA	−EDTA	+EDTA	−EDTA	+EDTA	−EDTA	+EDTA
SodB	66.7 ± 4.9	65.7 ± 5.9	65.3 ± 2.6	38.9 ± 3.3	67.8 ± 4.5	113.5 ± 10.0	71.1 ± 7.4	71.5 ± 7.0	65.2 ± 4.2	64.6 ± 7.8	62.2 ± 3.9
SodB-H28A	190.6 ± 3.0	NT	NT	171.5 ± 5.4	210.2 ± 0.2	NT	NT	NT	NT	NT	NT
SodB-D164A–H168A	No activity	NT	NT	No activity	No activity	NT	NT	NT	NT	NT	NT

NT, not tested.

Enzymatic activities of all variants of AB5075 SodB after Chelex 100 treatment were assayed using NBT as a substrate.

Each enzyme was assayed using two independent biological replicates, and error values quoted represent SD from the mean value given. Protein concentration ranges from 0 to 400 nM with equimolar concentration of metal ions. EDTA was used at a concentration of 50 mM to inhibit the metal-dependent activity of SOD, indicated as +, whereas the absence of EDTA is denoted as −. The enzymes' catalytic activities determined in terms of IC_50_ values were calculated using nonlinear regression.

To validate the growth suppression phenotype observed in the Δ*sodB* mutant strain, complementation was performed using pWBAD plasmid expressing the native *sodB* gene or its variants, *sodB*-H28A or *sodB*-D164A-H168A. The survival of these complemented strains under MV-induced oxidative stress elucidated the role of specific SodB residues in combating oxidative damage. Complementation with the native *sodB* successfully restored the growth of the Δ*sodB* strain to levels comparable to WT cells. In contrast, neither sodB-H28A nor sodB-D164A-H168A mutants were able to achieve similar restoration (Fig. 3*E*), underscoring the critical importance of these residues in thefunction of SodB. This result resonates with the evidence for Mn being important for oxidative stress response and possible metallation of Mn-SOD in *A. baumannii* (32). The expression of Mn-SOD is regulated by oxidative environments and is modulated both transcriptionally and post-translationally in a metal-dependent manner (33). Consistent with this observation, we detected a significant increase in the transcript abundance of *sodB* when AB5075 cells were exposed to oxidative stress in the presence of Mn^2+^. Conversely, supplementation with Fe^2+^ did not affect SodB expression level (Fig. S3*A*). These findings strongly suggest that AB5075 relies on Mn^2+^ as the essential cofactor, rather than Fe^2+^, for the efficient catalytic activity of SodB. Therefore, SodB in *A. baumannii* should be referred to as Mn-SOD.

### SodC in AB5075 utilizes Cu, not Zn, as a cofactor for optimal catalytic activity

**Figure 4:**
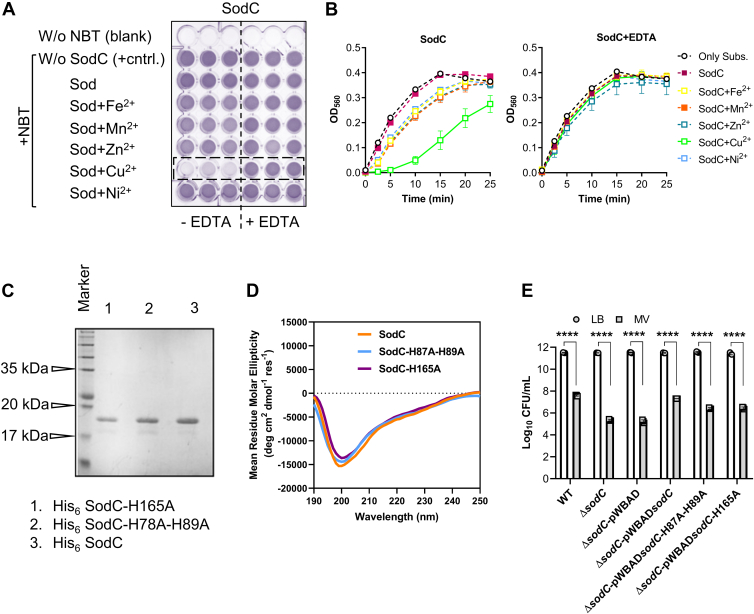
**SodC in AB5075 utilizes copper (Cu), not zinc (Zn) as a cofactor for optimal catalytic activity.***A,* AB5075 SodC (after the removal of the N-terminal 20 amino acids, which are signal peptides involved in protein export) was expressed in *Escherichia coli* BL21 (DE3) strain and FPLC purified followed by treatment with Chelex 100 to remove any bound metal ions. The activity of purified SodC was determined using NBT as a substrate. An equal amount of protein (50 nM) and an equimolar ratio of metal ions were used in this assay. EDTA was used at a concentration of 50 mM to inhibit the metal-dependent activity of SOD, indicated as +EDTA, whereas the absence of EDTA is denoted as −EDTA. The blank represents the protein in buffer without NBT added to the substrate buffer, whereas wells containing substrate buffer with NBT serve as a positive control without protein (+cntrl). The *dotted box* indicates the Cu-dependent efficient activity of SodC. *B,* SodC protein (50 nM) was incubated with the specified metal ions, with or without EDTA, and the formation of blue formazan was measured at an absorbance at 560 nm at the designated time points. Reactions containing substrate buffer with NBT serve as a positive control without protein (only subs.). *C,* SDS-PAGE analysis displaying the sizes of His-tagged (cleaved N-terminal 20 amino acids) native SodC, SodC-H87A–H89A, and SodC-H165A. *D,* overlaid CD spectra (n = 1) of His-tagged (cleaved N-terminal 20 amino acids) native SodC, SodC-H87A–H89A, and SodC-H165A, indicating similar secondary structure content among the three proteins. *E,* WT, ΔsodC, and ΔsodC complemented strains were grown to an absorbance of ∼0.6 at 600 nm in LB medium and pulsed with MV (250 μM) for 120 min. The data represent the mean of recovered CFU of three biological replicates, each in technical triplicates ± SD. Statistical significance was determined using a multiple comparison one-way ANOVA test with the Sidak correction for multiple comparisons, comparing the means of each group to one another. ∗∗∗∗ denotes *p* < 0.0001. CFU, colony-forming unit; MV, methyl viologen; NBT, nitroblue tetrazolium; SOD, superoxide dismutase; LB, lysogeny broth.

SodC is predominantly localized in the periplasmic space of Gram-negative bacteria, including notable pathogens, such as *Salmonella enterica*, *E. coli*, *Neisseria meningitidis*, *Haemophilus influenzae*, and *A. baumannii.* It plays a crucial role in protecting bacteria from ROS generated by the host immune system. By mitigating ROS-induced damage, SodC enhances bacterial survival and persistence within host tissues. Our findings demonstrate that SodC facilitates bacterial adhesion and invasion under host-induced oxidative stress, while also exhibiting a tissue-specific role in supporting bacterial survival during infection. In AB5075, SodC encodes a Cu/Zn-SOD, which presents a cofactor dilemma regarding its catalytic utility between Cu^2+^ and Zn^2+^ ions. To address this cofactor specificity, we conducted a comprehensive analysis of its metal-dependent catalytic activity. Unlike SodB, the crystal structure of SodC remains unresolved. Multiple sequence alignment of SodC proteins (Fig. S1*C*) revealed conservation of key catalytic residues across both bacterial species, indicating a preserved functional domain. The corresponding phylogenetic analysis (Fig. S1*D*) showed clear lineage-specific clustering, reflecting evolutionary relationships and confirming that SodC is conserved across a wide range of species. Metal ion preference analysis was performed for SodC to predict the binding affinities of Cu^2+^ and Zn^2+^, following the same approach used for SodB. The results indicated that SodC can bind to both Cu^2+^ and Zn^2+^, with binding scores of 4.785 and 5.475, respectively (Table S2). To further validate the metal-binding residues predicted by MIB for SodC, a similar approach to that used for SodB—employing AlphaFill and Schrödinger—was applied. The resulting metal coordination was in agreement with the MIB predictions, supporting the reliability of the identified binding residues in SodC. The native form of purified SodC exhibits a very low stoichiometric association with metal ions, such as Cu, Zn, Fe, Mn, and Ni, which further diminishes following Chelex-100 treatment. Upon reintroduction of these metal ions, a marked increase in metal binding is observed, as reflected by the elevated stoichiometric ratios (Fig. S4). *In vitro* studies using purified SodC demonstrated that its catalytic activity is exclusively dependent on Cu^2+^ (Table 2; Fig. 4, *A* and *B*), confirming a strong cofactor preference for Cu^2+^ over Zn^2+^. To further investigate the role of Cu^2+^-binding residues, we generated two site-directed mutants based on predicted Cu^2+^-binding pockets: SodC-H87A-H89A and SodC-H165A. Both mutants showed reduced binding scores for Cu^2+^ (Table S2), supporting the importance of these residues in mediating the catalytic activity of SodC. To assess the structural stability and catalytic activity of SodC mutants, we purified SodC-H87A-H89A and SodC-H165A mutant proteins and analyzed their sizes and structural characteristics using SDS-PAGE and CD spectroscopy. All purified proteins displayed comparable molecular sizes (Fig. 4*C*) and CD spectra (Fig. 4*D*) indicating no observable structural alterations. However, enzymatic activity assays revealed a significant reduction in the catalytic function of both mutants (Table 1), highlighting the critical role of these specific residues in the catalytic function of SodC.

**Table 2:** SOD activity of purified native SodC and mutant SodC proteins

Enzyme	Enzyme IC_50_ (nM)										
	Only	Fe		Mn		Zn		Cu		Ni	
	-	−EDTA	+EDTA	−EDTA	+EDTA	−EDTA	+EDTA	−EDTA	+EDTA	−EDTA	+EDTA
SodC	220.3 ± 9.7	131.2 ± 1.3	209.4 ± 7.5	149.9 ± 2.9	208.4 ± 5.7	155.7 ± 12.1	229.2 ± 15.5	42.1 ± 1.2	202.7 ± 6.6	145.5 ± 10.5	211.5 ± 7.3
SodC-H87A–H89A	156.8 ± 4.8	NT	NT	NT	NT	NT	NT	140.2 ± 9.1	167.5 ± 9.6	NT	NT
SodC-H165A	179.3 ± 11.5	NT	NT	NT	NT	NT	NT	172.5 ± 6.6	172.2 ± 2.9	NT	NT

NT, not tested.

Enzymatic activities of all variants of AB5075 SodC (cleaved N-terminal 20 amino acids) after Chelex 100 treatment were assayed using NBT as a substrate. Each enzyme was assayed using two independent biological replicates, and error values quoted represent SD from the mean value given. Protein concentration ranges from 0 to 400 nM with equimolar concentration of metal ions. EDTA was used at a concentration of 50 mM to inhibit the metal-dependent activity of SOD, indicated as +, whereas the absence of EDTA is denoted as −. The enzymes' catalytic activities determined in terms of IC_50_ values were calculated using nonlinear regression.

To validate the growth phenotype associated with the loss of the *sodC* gene, we complemented the Δ*sodC* strain using pWBAD plasmid encoding native *sodC*, *sodC*-H87A–H89A, or *sodC*-H165A. We then assessed the survival of these complemented strains under MV-induced oxidative stress. Complementation with native *sodC* successfully restored growth in the Δ*sodC* strain to levels comparable to the WT, whereas the mutants showed only partial restoration of growth (Fig. 4*E*). These results collectively demonstrate that AB5075 relies on Cu^2+^, rather than Zn^2+^, as an essential cofactor for the efficient catalytic activity of SodC, emphasizing the importance of Cu^2+^-binding residues in SodC functionality.

## Discussion

Metalloproteins are pervasive in biological systems and play vital roles in various cellular processes. Their activities are critically dependent on metal cofactors, with their utility and specificity shaped by intense selective pressures exerted at the host–pathogen interface. This evolutionary arms race drive host to develop strategies such as metal sequestration to restrict pathogen access to essential metals. In response, pathogens adapt by evolving subtle mechanisms to acquire and utilize preferred metal cofactors efficiently (34). In this study, we employed SODs from strain AB5075 as a model system to explore metal cofactor specificity for catalytic activity. We also investigated the roles of SodB and SodC in mitigating host-induced oxidative stress, providing insights into their functional preferences and contributions to bacterial survival during infection.

The metal cofactor utilized by a protein is not an immutable trait; it can adapt to environmental changes and selective pressures. During evolution, Fe was highly soluble and abundant in the anaerobic oceans, fostering the evolution of numerous Fe-utilizing enzymes and organisms (35). However, the oxygenation of the atmosphere because of early photosynthetic organisms decreased the availability of Fe, resulting in selective pressures that prompted the adaptation of Fe-dependent enzymes to utilize alternative cofactors (33, 36, 37). In *E. coli*, Fe-dependent Fe-SOD demonstrates the ability to switch from Fe^2+^ as a cofactor to Mn^2+^ under specific conditions, showcasing its adaptability to environmental metal availability. Comparative analyses of Fe-SODs and Mn-SODs have revealed the striking structural similarities, particularly in the active site residues located at the dimer interface. These residues form a funnel-like structure that guides the superoxide substrate toward the catalytic metal ion (38). The reduction midpoint potential (Em) is a thermodynamic parameter that indicates the tendency of a metal ion in a metalloprotein to gain electrons during a redox reaction. The lower Em value denotes a greater stability of metal ions and efficient catalytic activity. The stronger hydrogen bond donation observed in Mn-SOD accounts for the lowered *Em* (reduction midpoint potential) associated with Mn-SOD (38). This suggests a probable mechanism by which evolution optimized the SOD protein for function with Mn^2+^ while making relatively minor structural changes without affecting the residues directly responsible for binding the metal ion (39). *Borrelia burgdorferi* possesses a single Mn-SodA and lacks Fe-SodB, reflecting a strategic adaptation to the limited Fe supplies in host environments (39, 40). In some instances, such as *Salmonella* Typhimurium, the absence of SodA does not affect virulence (41). In contrast, the loss of intracellular SODs in organisms like *Streptococcus agalactiae* and *B. burgdorferi* diminishes virulence, despite no significant growth inhibition under laboratory conditions (42, 43). Our study provides experimental evidences that *A. baumannii* 5075 preferentially utilizes SodB to mitigate oxidative stress imposed by the host. This raises the question: how can an intracellular SOD enhance virulence when host-derived superoxide is predominantly extracellular? While superoxide radicals typically does not permeate biological membranes, can cross them when protonated (44). In acidic environments such as the macrophage phagolysosome, protonated superoxide may diffuse into bacterial cells, where it can serve as a substrate for intracellular SodA and SodB (44, 45). These intracellular SODs then play a critical role in protecting bacteria from the oxidative damage caused by internally generated superoxide, thereby contributing to bacterial survival and fitness during infection (44). In contrast, the extracellular Cu/Zn-SOD, SodC, has been shown to interact with host superoxide and is recognized as an important virulence factor in several pathogenic bacteria (46). Notably, our results revealed that the deletion of *sodB* resulted in a more pronounced attenuation of virulence compared with the deletion of *sodC*, highlighting the critical role of SodB in counteracting host-induced oxidative stress and supporting pathogenicity.

Historically, it was believed that all Cu-containing SODs also require Zn as a cofactor (44). However, a notable exception exists in the form of SodC from *Mycobacterium tuberculosis* and its closely related species, including *Mycobacterium leprae* and *Mycobacterium avium*. This enzyme functions as a Cu-only SOD, exhibiting enzymatic activity in the absence of Zn (47). Similarly, we observed that SodC in AB5075 utilizes Cu^2+^ for its efficient catalytic activity, whereas the presence of Zn^2+^ does not contribute to its function (Fig. 4). The Cu-exclusive nature of SodC provides a significant advantage in environments where Zn availability is restricted, particularly in the context of host-mediated “nutritional immunity.” During such immune responses, the host actively sequesters free Zn to create a Zn-starved environment that hampers the growth and survival of invading bacterial pathogens (12, 48). In this scenario, the strategy of Zn sequestration would be ineffective against a Cu-only SOD-like SodC, which does not rely on Zn ion for its catalytic activity. This characteristic promotes the survival of bacteria in host tissues, allowing them to withstand the host's attempts to limit their access to essential nutrients.

This study demonstrates that the putative Fe-SOD utilizes only Mn^2+^ ion, whereas the putative Cu/Zn-SOD relies solely on Cu^2+^ ion as a cofactor for catalytic activity. Our findings unequivocally redefine the annotation of SodB, transitioning its classification from Fe-SOD to Mn-SOD because of its exclusive dependence on Mn as the sole catalytic cofactor. In conclusion, our findings emphasize the remarkable adaptability of SODs to utilize metal ion cofactors for catalytic activity and highlight their significance in bacterial virulence. The ability of these enzymes to selectively utilize different metal cofactors in response to environmental changes reflects a crucial evolutionary strategy that enhances the survival of bacteria in diverse niches. Future research should focus on elucidating the underlying mechanisms governing metal cofactor selection and the biochemical pathways that facilitate this adaptability. Understanding these processes could reveal critical insights into how bacteria optimize their metabolic functions and cope with host-induced stressors (37). Furthermore, investigating the implications of metal cofactor dynamics on bacterial survival and pathogenicity may inform the development of novel therapeutic strategies to target metalloenzyme functions in pathogenic bacteria, ultimately contributing to more effective approaches in combating bacterial infections (49).

## Experimental procedures

### Bacterial strains and growth conditions

Bacterial strains and plasmids used in this study are listed in Tables S5 and S6. *A. baumannii* and *E. coli* strains were grown at 37 °C in LB broth or LB agar. *A. baumannii* strains that contain the pWBAD30 plasmid with Apramycin^R^ cassette were grown in LB broth or LB agar, supplemented with 30 μg/ml apramycin.

### Bacterial growth assay under oxidative stress

Midlog phase cultures of WT5075, Δ*sodB*, and Δ*sodC* strains were inoculated at 0.1% into 200 μl of fresh LB medium containing 150 μM MV. Growth assays were conducted in sterile 96-well plates (Genaxy) at 37 °C with shaking at 20 CPM, measuring absorbance at 600 nm every 30 min for the specified duration using a Synergy microplate reader (BioTek). Media without any culture served as a negative control. The data presented are after background correction.

### Intracellular ROS level determination

Bacterial cells were grown at 37 °C with shaking to an absorbance of 0.6 at 600 nm (midlog phase) in LB medium. After centrifugation, the cells were washed with sterile 1X PBS and resuspended in the same buffer. A final concentration of 100 μM 2′,7′-dichlorofluorescein diacetate (Thermo Fisher Scientific; D399) was added, and the cells were incubated for 30 min at 37 °C. The cells were washed with 1X PBS to remove excess dye and transferred to a 96-well transparent black plate (BRAND) with 100 μl per well. MV was added at a final concentration of 250 μM, and the samples were incubated at 37 °C with shaking at 20 RPM. Absorbance at 600 nm and fluorescence (excitation/emission at 485/535 nm) were measured every 10 min using a Synergy microplate reader. The presented data have been background-corrected and normalized to an absorbance at 600 nm.

### Estimation of bacterial colony-forming unit under oxidative stress

Bacterial cells were grown at 37 °C with shaking to an absorbance of 0.6 at 600 nm (midlog phase) in LB medium. MV was added to a final concentration of 250 μM, and the samples were incubated at 37 °C with shaking at 180 RPM for an additional 2 h. Meanwhile, VitC was added at a final 0.5 mg/ml concentration. The bacterial cells were then diluted and plated on LB agar to count the colony-forming unit (CFU).

### Quantitative RT–PCR analysis

Bacterial cells were grown at 37 °C with shaking to an absorbance of 0.6 at 600 nm (midlog phase) in LB medium. MV was added to a final concentration of 250 μM, and the samples were incubated at 37 °C with shaking at 180 RPM for an additional 2 h. The bacterial cells were harvested by centrifugation at maximum speed. After washing the cell pellet with 1X PBS, RNA was extracted from the bacterial cells by the classic phenol–chloroform method. The complementary DNA synthesis was performed using PrimeScript first strand cDNA Synthesis Kit (TakaRa; 28704) according to the manufacturer's instructions. Amplifications were achieved using a three-step program on a QuantStudio 5 system (Thermo Fisher Scientific). The transcript abundance was calculated using the ΔΔC_T_ method and normalized by the 16s gene.

### NBT assay with bacterial cell lysate to determine SOD activity

SOD activity was assessed using NBT as a substrate. Bacterial cultures were grown in LB-medium till an absorbance of ∼0.6 at 600 nm was achieved, MV was added at a final concentration of 250 μM and grown for another 2 h. After an absorbance at 600 nm normalization, bacterial cells were harvested and lysed the bacterial pellet. The cell lysates at an equal amount were incubated with a buffer containing 50 mM potassium phosphate, 14 mM *N*,*N*,*N*',*N*'-tetramethylethylenediamine, 10 μM riboflavin, and 50 μM NBT. The samples were then exposed to white light for 5 to 10 min. The presence of active SOD inhibits the photochemical reduction of NBT in the presence of riboflavin, which is visually observable. The absorbance at 560 nm was measured, and the percentage reduction in an absorbance at 560 nm was calculated to reflect SOD activity.

### Bacterial adhesion and invasion assay

For adhesion and invasion assays, A549 cells were seeded in 24-well tissue culture plates at a density of 1 × 10^5^ cells per well. After 24 h of incubation in an antibiotic-free medium, midlog phase bacterial cells were added at a multiplicity of infection of 100 in Dulbecco's modified Eagle's medium supplemented with 1% fetal bovine serum and incubated for 2 h at 37 °C in 5% CO_2_. After removing nonadherent bacteria through washing, cells were lysed with 0.1% Triton X-100 (Sigma), and serial dilutions of the suspension were plated onto LB agar to determine bacterial CFU. To assess the number of intracellular bacteria, the infected A549 cells were treated with gentamicin (300 μg/ml) for 2 h at 37 °C. After washing, the cells were lysed, and the recovered bacteria were plated onto LB agar to calculate bacterial CFU.

### Biofilm formation assay

Bacterial cells were grown at 37 °C with shaking to an absorbance of 0.6 at 600 nm (midlog phase) in LB medium. A 1% inoculum from each culture was transferred into fresh LB medium in a 12-well plate, followed by incubation at 37°C for 24 h. Following incubation, the absorbance at 600 nm of the cell suspensions was measured. The tubes were then washed extensively with sterile water and air-dried. Subsequently, 1% crystal violet solution was added to each well and incubated at room temperature for 30 min. After incubation, the tubes were again washed with sterile water. The bound crystal violet stain was solubilized using 30% acetic acid in water, and the absorbance at 595 nm was recorded. The ratio of absorbance at 595 nm to absorbance at 600 nm was calculated for each well. Wells containing only LB medium were used as a negative control.

### Metal ion–binding prediction and structural validation

Metal ion–binding sites in SodB and SodC were initially predicted using the Metal Ion–Binding Site Prediction and Modeling Server (MIB), wherein Fe^2+^ and Mn^2+^ were docked to SodB, and Zn^2+^ and Cu^2+^ to SodC, including their respective mutant variants. Predicted binding residues and MIB scores were documented for each protein–ion complex. To validate and refine these predictions, new models were generated and further assessed using AlphaFill and the Schrödinger software suite. Protein structures corresponding to UniProt IDs B0VUT7 (*sodB_AB5075*) and *A0A0D8G9Q7* (*sodC_AB5075*) were retrieved and loaded into AlphaFill, where the respective metal ions were automatically detected and modeled. The resulting 3D structures were then imported into Schrödinger for further processing. Protein structures were prepared using the Protein Preparation Wizard, which included assignment of bond orders, addition of hydrogens, optimization of hydrogen bonding networks, and correction of ionization states. This was followed by macromodel-based energy minimization to resolve steric clashes and optimize local geometry. Finally, metal ion and active site residue interactions were analyzed to confirm coordination feasibility and structural consistency compared with MIB models.

### Cloning, expression, and purification of recombinant SodB and its mutants

The expression construct of *A. baumannii* SodB was prepared by using primers listed in Table S7 with NheI/XhoI restriction cloning into pET28a vector in *E. coli* DH5α cells (Invitrogen). The SodB expression vector (pET28a-*sodB* vector) was transformed into *E. coli* BL21 (DE3) cells (Invitrogen). Freshly transformed cells were grown in LB medium supplemented with kanamycin (50 μg/ml) at 37 °C until an absorbance of 0.6 at 600 nm. Protein expression was induced with 0.5 mM IPTG, and the cells were grown at 37 °C for an additional 3.5 h at 180 RPM. Cells were harvested by centrifugation and resuspended in buffer A (50 mM Tris–HCl, pH 7.3, 500 mM NaCl, 1 mM β-mercaptoethanol, and 10% glycerol). Cells were lysed by French press, followed by centrifugation at 10,000 RPM at 37 °C. Cleared cell lysate was subjected to chromatographic separation using an AKTA purification system (GE Healthcare). Recombinant SodB proteins were purified using affinity chromatography (HisTrap FF column; GE Healthcare) in 50 mM Tris–HCl (pH 7.3), 500 mM NaCl, 1 mM β-mercaptoethanol, 10% glycerol buffer with 50 to 500 mM imidazole gradient elution, and subsequently by size-exclusion chromatography in 20 mM Tris–HCl, 200 mM NaCl (pH 7.3) buffer on a HiLoad 16/600 Superdex 200 pg column (GE Healthcare). The constructs for recombinant expression of SodB mutants were generated using the site-directed mutagenesis using In-fusion kit (TaKaRa; catalog no.: 638948), using the pET28a-*SodB* vector. All the native and mutant constructs were confirmed by sequencing. The purified proteins were treated with Chelex 100 resin to remove bound metal. The Chelex 100 treatment was confirmed by assessing the enzyme activity with or without EDTA. The purified protein fractions were pooled and concentrated using an Amicon filter (Merck). The bicinchoninic acid (BCA) method (BCA kit; Takara) determined the concentration of recombinant proteins with BSA as a standard.

### Cloning, expression, and purification of recombinant SodC and its mutants

The cloning of the native and mutant SodCs into pET28a vector in *E. coli* DH5α cells (Invitrogen), their expression in *E. coli* BL21 (DE3) cells, and the purification of the recombinant proteins were performed as described previously. The recombinant SodC native and mutant proteins used for *in vitro* studies are devoid of N-terminal 20 amino acid–long signal peptide. The recombinant SodC native and mutant proteins were further purified by cation-exchange chromatography (HiTrap Heparin HP column; GE Healthcare) in 50 mM Tris–HCl (pH 7.3), 10% glycerol, 1 mM β-mercaptoethanol buffer with 10 to 500 mM NaCl gradient elution after affinity-based purification. The constructs for recombinant expression of SodC mutants were generated as described previously, using the pET28a-*sodC* vector. Chelex 100 treatment was confirmed by assessing the enzyme activity with or without EDTA. All the native and mutant constructs were confirmed by sequencing. The purified protein fractions were pooled and concentrated using an Amicon filter (Merck). The BCA method (BCA kit; Takara) determined the concentration of recombinant proteins with BSA as a standard.

### Chelex treatment, remetallation, gel filtration, and inductively coupled plasma mass spectrometry analysis of SOD proteins

To obtain the apo (metal-depleted) forms of SodB and SodC, the native purified SOD proteins at an equal concentration were treated with Chelex-100 resin. To reconstitute Chelex-treated metal-depleted SodB and SodC proteins, samples were incubated with 1:10 M ratio of metal ions (Mn^2+^, Ni^2+,^ Cu^2+^, Zn^2+^, and Fe^2+^) in metal-free 20 mM Tris–HCl, 200 mM NaCl (pH 7.3) at 37 °C for 1 h. After incubation, excess unbound metal ions were removed using a GE Healthcare NAP-10 Sephadex G-25 column under the same buffer conditions. This gel filtration step ensured the separation of protein-bound metal from free metal ions in solution. To assess the identity and stoichiometry of metal ions bound to SodB and SodC in their native, Chelex-treated, and remetallated states, protein samples were analyzed by inductively coupled plasma mass spectrometry (ICP-MS). Prior to analysis, protein concentrations were determined by BCA Protein assay Kit using the manufacturer's protocol. Equimolar concentration of SOD proteins were subjected to digestion with 3% (v/v) trace-metal grade nitric acid (HNO_3_) and diluted to a final volume of 5 ml. Samples were analyzed using an Agilent Model- 8900 ICP-MS Triple Quad, calibrated with certified multielement standards. Background correction was performed using buffer-only controls. The molar concentration of each metal ion detected by ICP-MS (He atom) was calculated by their individual molecular weight of the metals. The calculation of metal-to-protein stoichiometric ratio molar concentration was done by comparing the metal-to-protein molar concentration. The resulting ratio reflects the number of metal ions bound per molecule of protein, enabling a direct comparison of metallation status across native, Chelex-treated, and reconstituted conditions.

### Determination of enzyme activity of purified SOD proteins

The purified SOD proteins at an equal concentration after Chelex 100 treatment were incubated with or without equimolar concentration of indicated metals in the presence or the absence of EDTA (50 nM) at 37 °C for 15 min. A substrate buffer containing 50 mM potassium phosphate, 14 mM *N*,*N*,*N*',*N*'-tetramethylethylenediamine, 10 μM riboflavin, and 50 μM NBT was added to the samples. The samples were then exposed to white light for 15 min. The presence of active SOD inhibits the photochemical reduction of NBT in the presence of riboflavin, which is visually observable. The absorbance was measured at 560 nm, and the IC_50_ value was calculated from a nonlinear regression analysis. The plate was exposed to white light for kinetic experiments, and an absorbance at 560 nm was measured at the indicated time points.

### CD spectroscopy

CD spectra were collected using J-1500 CD Spectrometer (JASCO) in the far-ultraviolet range (190–250 nm) with a scan rate of 100 nm/min, a bandwidth of 1 nm, and a data pitch of 1 nm. Spectral measurements were performed at a protein concentration of 0.3 mg/ml for SodB native and mutant proteins and 0.1 mg/ml for SodC native and mutant proteins in 20 mM sodium phosphate buffer (pH 7.3) at 25 °C in a 1 mm path length quartz cuvette (Hellma). Each spectrum is the average of three scans corrected for the buffer reference. The comparison of WT and mutant SOD proteins was made by calculating the mean residue molar ellipticity (deg cm^2^ dmol^-1^ res^-1^) using the formula:

$$\frac{Ellipticity\;\left(mdeg\right)\;.\;10^{6}}{Path\;length\;\left(mm\right)\;.\;Protein\;concentration\;\left(\mu M\right)\;.\;N}$$

N = Number of amino acid residues in protein.

To evaluate the secondary structure content, the CD spectra data of WT and mutant forms of SodB and SodC were analyzed using a web server BeStSel, designed for accurate protein secondary structure prediction and fold recognition from CD spectra.

### Estimation of bacterial CFU of mutants under oxidative stress

Bacterial cells were grown at 37 °C with shaking to an absorbance of 1.0 at 600 nm in LB medium. MV was added to a final concentration of 250 μM followed by the addition of _L_-arabinose (final concentration 0.2% w/v), and the samples were incubated at 37 °C with shaking at 180 RPM for an additional 2 h. _L_-arabinose was used to induce the *SOD* expression of complemented strains. The bacterial cells were then diluted and plated on LB agar to count the CFU.

### Mouse ROMO1 (ROS modulator)–based ELISA

All animal experiments conducted under protocol BT/IAEC/2020/02 were reviewed and approved by the Institutional Animal Ethics Committee at the Indian Institute of Technology, Roorkee. Male C57BL/6J mice (4–6 weeks old, n = 4) were procured from IMTECH, Chandigarh and quarantined for 1 week before experimentation. Mice were fed an HFD (60% fat treatment diet) continuously for 14 days. Group 1 received a normal-fed diet, group 2 was given an HFD (60% fat), and group 3 was administered an HFD along with *N*-acetyl l-cysteine (ROS quencher) in drinking water. All groups of mice were euthanized, and lung and liver tissues were harvested under sterile conditions. The tissues are immediately placed on ice and washed with ice-cold 1X sterile PBS and homogenized. ROS levels were quantified in the three groups using the FineTest Mouse ROMO1 ELISA Kit (catalog no.: EM0699) following the manufacturer's protocol.

### Mice infection model for AB5075 systemic infection

All animal experiments conducted under protocol BT/IAEC/2020/02 were reviewed and approved by the Institutional Animal Ethics Committee at the Indian Institute of Technology, Roorkee. Four- to 6-week-old male C57BL/6J mice (n = 7) were procured from IMTECH, Chandigarh and quarantined for 1 week before experimentation. Mice were fed an HFD (60% fat treatment diet) continuously for 14 days. To investigate the role of ROS scavengers, one group of mice was given NAC (Himedia; RM578) at 100 mg/l in their drinking water throughout the experiment. Mice were anesthetized and systemically infected with a bacterial inoculum containing 4 × 10^4^ CFU of the specified strains. At 36 h postinfection, the mice were euthanized, and the lungs and livers were harvested. These organs were immediately placed on ice, washed with ice-cold 1X sterile PBS, homogenized, and then plated onto leeds *Acinetobacter* agar medium to enumerate the bacterial burden.

### Statistical analysis

Statistical analyses were conducted using GraphPad Prism 8 (GraphPad Software, Inc). Details regarding the number of repeated experiments, statistical tests employed, significance values, and group sizes are provided in each figure legend.

## Data availability

All data are included in the article or the supporting information. Any additional information required to analyze the data reported in this article is available upon request to the corresponding author.

## Supporting information

This article contains supporting information (Figs. S1–S4 and Tables S1–S7).

## Conflict of interest

The authors declare that they have no conflicts of interest with the contents of this article.
