# Supplementary material for: Manganese-dependent iron-superoxide dismutase drives Acinetobacter baumannii fitness during oxidative stress
Source: J Biol Chem. 2025 Aug 5;301(9):110549. doi: 10.1016/j.jbc.2025.110549 (PMC12398933; doi:10.1016/j.jbc.2025.110549)
Supplement: Supporting Information [file mmc1.docx]

**Supporting Information**

**Manganese-dependent iron-superoxide dismutase drives *Acinetobacter baumannii* fitness during oxidative stress**

Ashish Kumar Ray^1#^, Somok Bhowmik^1#^ , Snehlata Saini^1#^, Arsalan Hussain^1^, Perwez Bakht^1^ , Shivam Pandey^1^, Ranjana Pathania^1,2,*^

^1^Department of Biosciences and Bioengineering, Indian Institute of Technology Roorkee, Uttarakhand – 247667, India

^2^Centre of Excellence in Disaster Mitigation and Management, Indian Institute of Technology Roorkee, Uttarakhand – 247667, India

^*^To whom correspondence should be addressed. E-mail: ranjana.pathania@bt.iitr.ac.in

# Equal Contribution

**Supporting Figures**

**
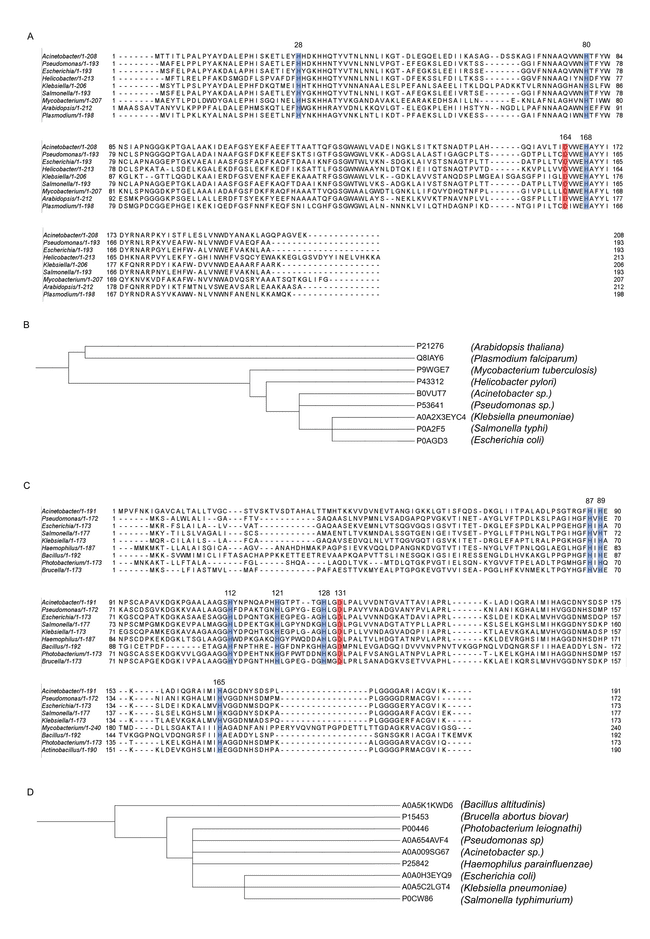
**

**S1. Multiple sequence alignments and phylogenetic analysis of conserved domains in SOD proteins across different bacterial and eukaryotic species.** (A, C) Multiple sequence alignments of SodB and SodC protein sequences from various organisms, showing conservation of key amino acid residues. Highly conserved histidine residues are shaded in blue, while functionally or structurally important residues (aspartate) are highlighted in red. Gaps introduced for alignment are indicated by dashes (–). Amino acid positions corresponding to important motifs or conserved domains are numbered above the sequences. (B, D) Phylogenetic trees constructed using the Interactive Tree of Life (iTOL) platform based on the sequence alignments shown in panels A and C, respectively. The tree was generated using an appropriate evolutionary algorithm (e.g., Neighbor-Joining or Maximum Likelihood) to infer evolutionary relationships. Protein accession numbers are shown alongside species names. The topology of the tree reflects evolutionary distances and sequence similarity among homologous proteins across the included species.***
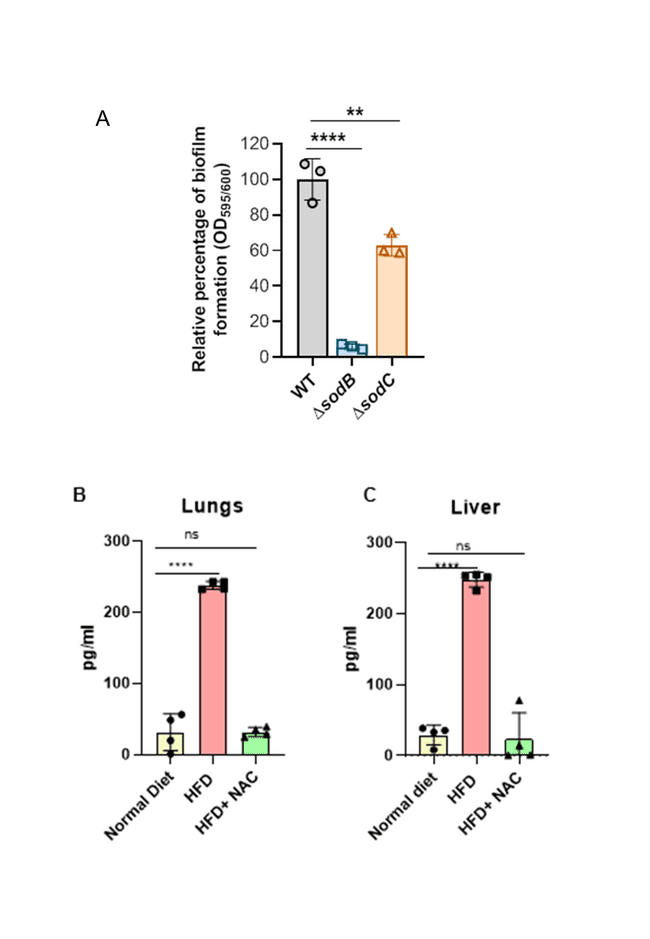
***

**S2. (A)** The relative percentage of biofilm formation in WT-5075, ∆*sodB*, and ∆*sodC* strains were determined. Data plotted after blank normalization. The data represent the mean ± SD. Statistical significance was determined using the one-way ANOVA test with Tukey’s multiple comparisons. ** denotes p-value <0.01, **** denotes p-value <0.0001. **(B-C)** The concentration of ROMO1(ROS Modulator 1) in the samples was estimated in three groups 1. Normal diet, group 2. High-fat diet, group 3. High-fat diet + NAC ( ROS quencher) in lungs and liver respectively. The data represent the mean ± SD. Statistical significance was determined using the one-way ANOVA test with Tukey’s multiple comparisons. ns denotes p-value >0.9, **** denotes p-value <0.0001.

**
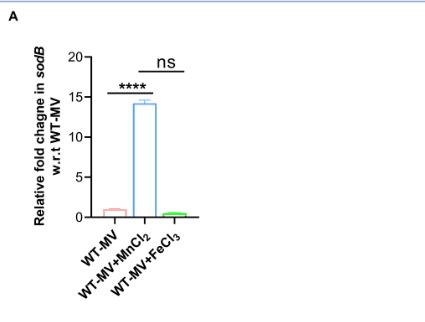
**

**S3.** The transcript levels of *sodB* were determined in WT-5075 cells grown in LB-medium to OD_600_~0.6 and pulsed with MV (250 µM) for 120 min in the presence of either MnCl_2_ or FeCl_3_ by qRT-PCR. The data represent the mean ± SD. Statistical significance was determined using the one-way ANOVA test with Tukey’s multiple comparisons. **** denotes p-value <0.0001, ns denotes not significant.


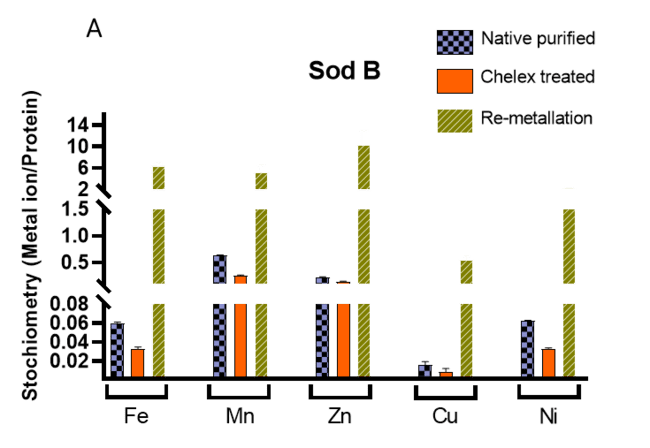


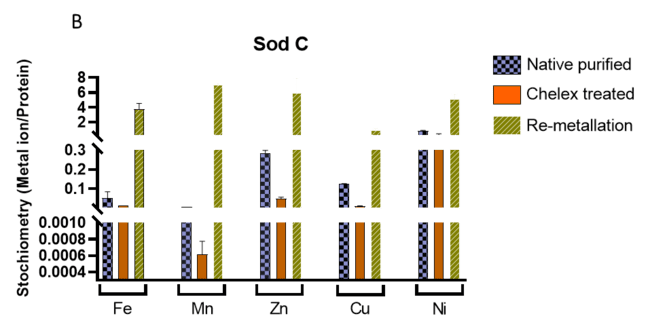


***S4. A-B***, Metal ion stoichiometry of SodB and SodC under native, Chelex-treated, and re-metallated conditions. Bar graphs represent the molar ratio of individual metal ions (Mn²⁺, Ni²⁺, Cu²⁺, Zn²⁺, and Fe²⁺) bound per molecule of SodB or SodC protein in three experimental states: native purified, Chelex-100 treated (metal-depleted), and re-metallate

**Supplementary Tables**

**Table S1. The binding residues of SodB predicted to interact with Fe and Mn were identified using the MIB web server, AlphaFill, and Schrödinger**

| **Protein** | **Metal ion** | **Docked model (MIB)** | **Predicted Binding residues (MIB)** | **Score**  **(MIB)** | **Modeling using AlphaFill and Schrödinger** |
| --- | --- | --- | --- | --- | --- |
| SodB | Fe^2+^ | **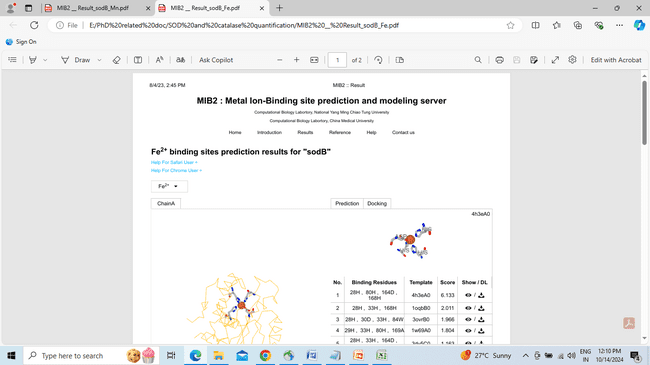** | 28H,80H,  164D,168H | 6.133 | **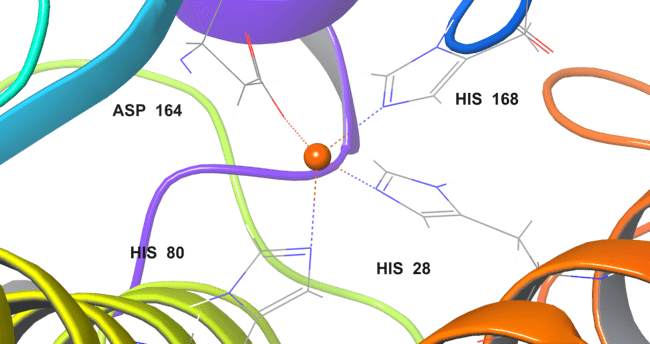** |
| SodB | Mn^2+^ | **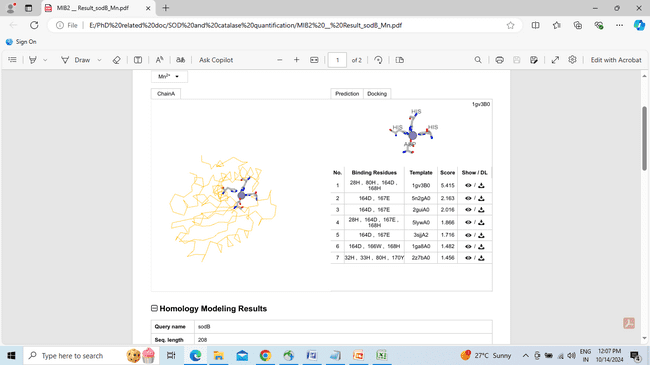** | 28H,80H,  164D,168H | 5.415 | **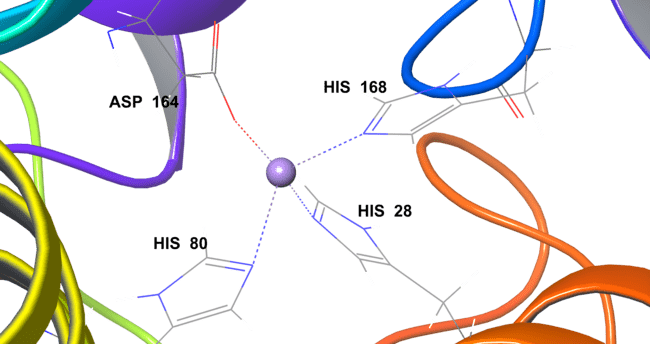** |
| SodB-H28A | Mn^2+^ | **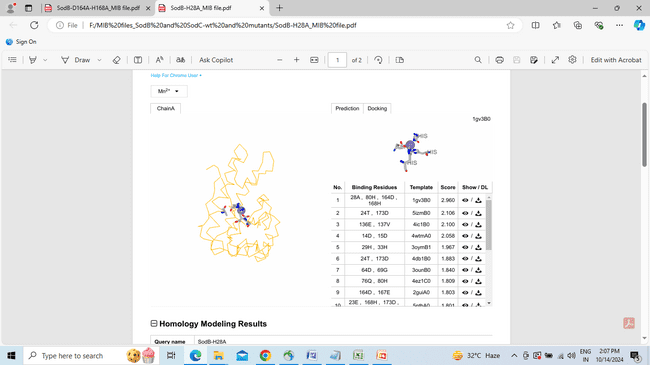** | 28A,80H,  164D,168H | 2.960 | **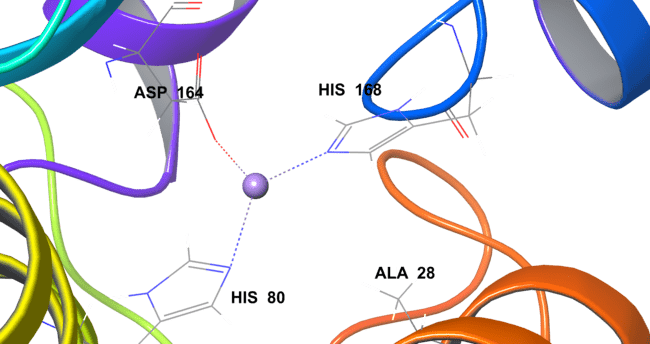** |
| SodB-D164A-H168A | Mn^2+^ | **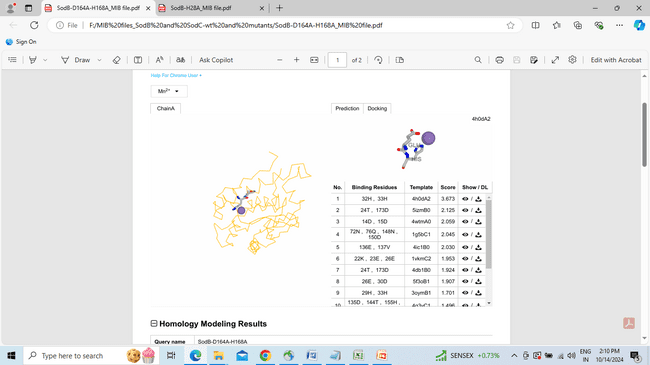** | 32H,33H | No binding | **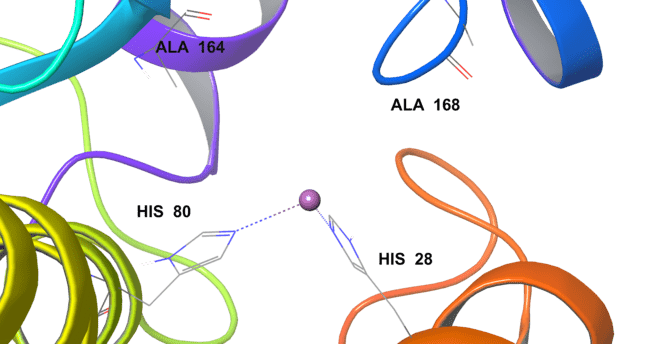** |

**Table S2. The binding residues of SodC predicted to interact with Zn and Cu were identified using the MIB web server, AlphaFill, and Schrödinger.**

| **Protein** | **Metal ion** | **Docked model (MIB)** | **Predicted Binding residues (MIB)** | **Score**  **(MIB)** | **Modeling using AlphaFill and Schrödinger** |
| --- | --- | --- | --- | --- | --- |
| SodC | Zn^2+^ | **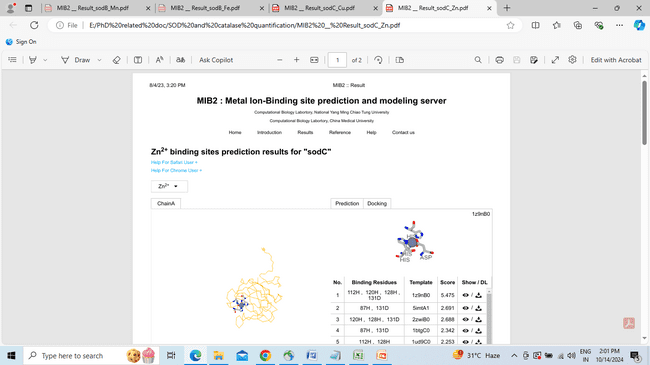** | 112H,120H,128H,131D | 5.475 | **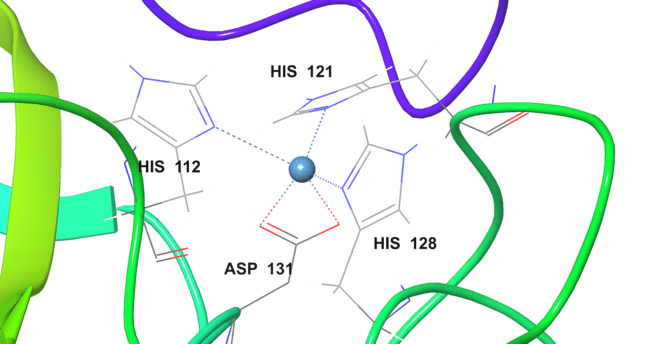** |
| SodC | Cu^2+^ | **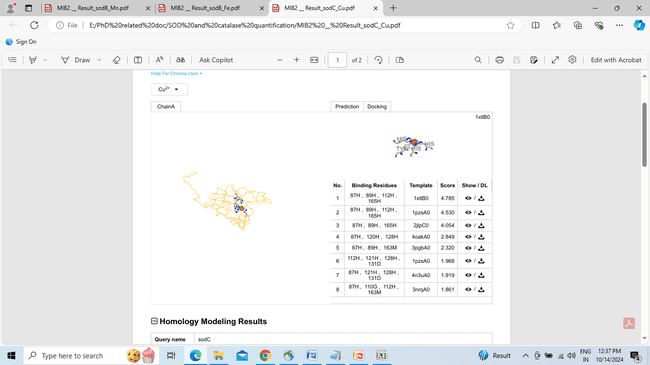** | 87H,89H, 112H,165H | 4.785 | **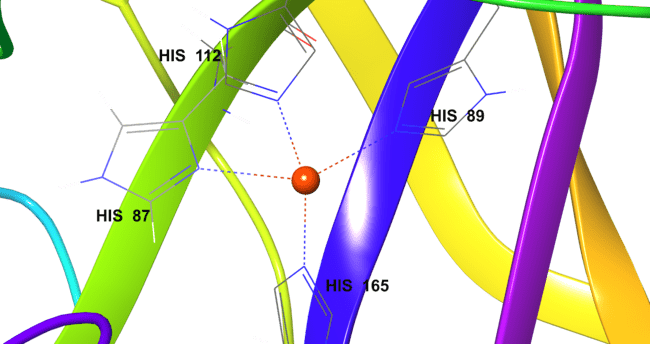** |
| SodC-H87A-H89A | Cu^2+^ | **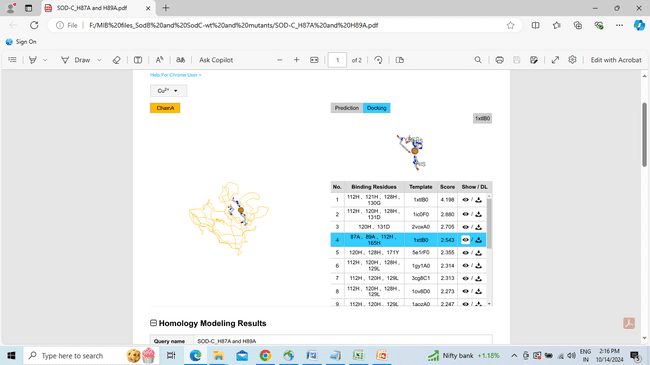** | 87A,89A, 112H,165H | 2.543 | **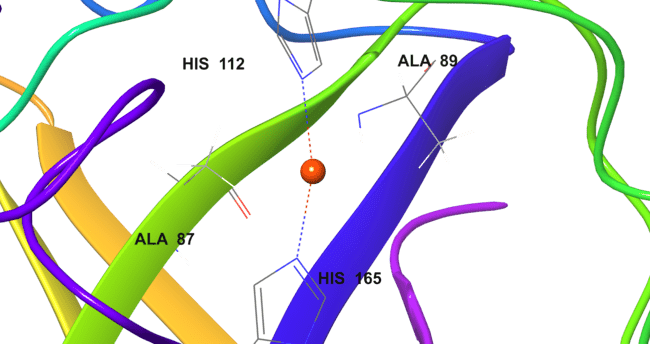** |
| SodC-H165A | Cu^2+^ | **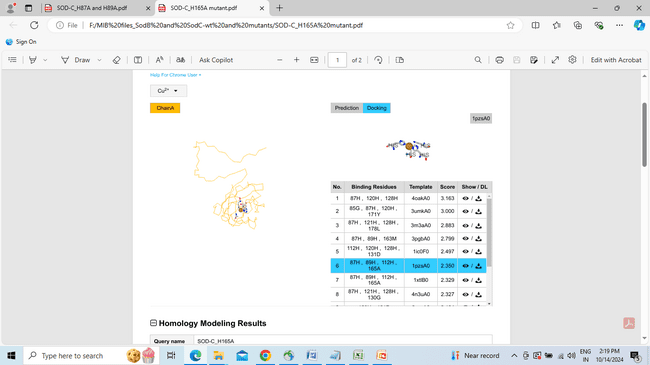** | 87H,89H, 112H,165A | 2.350 | **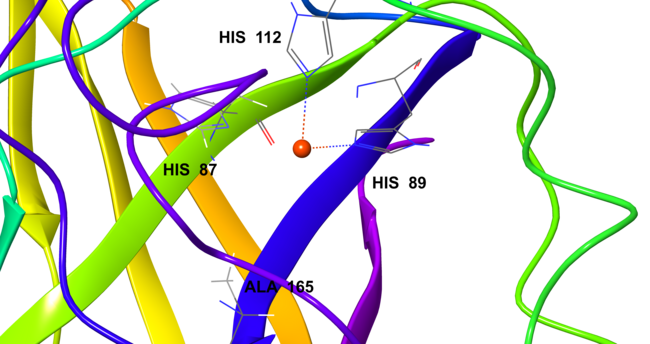** |

| **Protein** | **Helix** | **Antiparallel** | **Parallel** | **Turn** | **Others** |
| --- | --- | --- | --- | --- | --- |
| SodB | 34.1 | 8.6 | 0.0 | 9.8 | 47.5 |
| SodB-H28A | 32.7 | 11.6 | 4.5 | 8.6 | 42.6 |
| SodB-D164A-H168A | 29.2 | 14.6 | 0.0 | 10.4 | 45.8 |

**Table S3:** Estimated secondary structure content (%) of SodB-wild type and mutant proteins using BeStSel software.

**Table S4:** Estimated secondary structure content (%) of SodC-wild type and mutant proteins using BeStSel software.

| **Protein** | **Helix** | **Antiparallel** | **Parallel** | **Turn** | **Others** |
| --- | --- | --- | --- | --- | --- |
| SodC | 10.4 | 22.2 | 6.2 | 15.7 | 45.5 |
| SodC-H87A-H89A | 6.2 | 22.6 | 6.0 | 14.6 | 50.4 |
| SodC-H165A | 6.1 | 24.9 | 5.4 | 16.2 | 47.4 |

**Table S5.** List of bacterial strains used in this study.

| **Strains** | **Name** | **Description** | **Source** |
| --- | --- | --- | --- |
| *Acinetobacter baumannii* 5075 strain | AB5075 | Wild-type strain | Purchased from Manoil lab, University of Washington, Seattle |
| ∆*sodB A. baumannii* 5075 | ∆*sodB* | *sodB* mutant in *A. baumannii* 5075 strain, AB03297 | Purchased from Manoil lab, University of Washington, Seattle |
| ∆*sodC A. baumannii* 5075 | ∆*sodC* | *sodC* mutant in *A. baumannii* 5075 strain, AB00947 | Purchased from Manoil lab, University of Washington, Seattle |
| *Acinetobacter baumannii* ATCC 17978 | AB17978 | Wild-type strain | Purchased from ATCC, USA |
| *E. coli* DH5α carrying pET28a-*sodB* (native) | *E. coli* DH5α- pET28a-*sodB* (native) | *E. coli* DH5α carrying plasmid construct pET28a-*sodB* AB5075 SodB (native), Kan^R^ | This study |
| *E. coli* DH5α carrying pET28a-*sodB-*H28A mutant | *E. coli* DH5α- pET28a-*sodB-*H28A | *E. coli* DH5α carrying plasmid construct pET28a-*sodB-*H28A mutant, single mutation at 28^th^ amino acid position (Histidine to Alanine) AB5075 SodB-H28A mutant, Kan^R^ | This study |
| *E. coli* DH5α carrying pET28a-*sodB-*D164A-H168A mutant | *E. coli* DH5α- pET28a-*sodB-* D164A-H168A | *E. coli* DH5α carrying plasmid construct pET28a-*sodB-*D164A-H168A mutant, double mutation at 164^th^ and 168^th^ amino acid positions (Aspartate to Alanine and Histidine to Alanine, respectively) AB5075 SodB-D164A-H168A mutant, Kan^R^ | This study |
| *E. coli* DH5α carrying pET28a-*sodC* (native) without signal peptide | *E. coli* DH5α- pET28a-*sodC* (native) | *E. coli* DH5α carrying plasmid construct pET28a-*sodC* AB5075 SodC (native), Kan^R^ | This study |
| *E. coli* DH5α carrying pET28a-*sodC-*H87A-H89A mutant without signal peptide | *E. coli* DH5α- pET28a-*sodC-*H87A-H89A | *E. coli* DH5α carrying plasmid construct pET28a-*sodC-*H87A-H89A mutant, double mutation at 87^th^ and 89^th^ amino acid positions (Histidine to Alanine) AB5075 SodC-H87A-H89A mutant, Kan^R^ | This study |
| *E. coli* DH5α carrying pET28a-*sodC-*H165A mutant without signal peptide | *E. coli* DH5α- pET28a-*sodC-*H165A | *E. coli* DH5α carrying plasmid construct pET28a-*sodB-*H165A mutant, single mutation at 165^th^ amino acid position (Histidine to Alanine) AB5075 SodB- H165A mutant, Kan^R^ | This study |
| *E. coli* DH5α carrying pWBAD30-*sodB* (native) | *E. coli* DH5α- pWBAD30-*sodB* | *E. coli* DH5α carrying plasmid construct pWBAD30-*SodB* (native) for complementation, Apr^R^, Kan^R^ | This study |
| *E. coli* DH5α carrying pWBAD30-*sodB*-H28A mutant | *E. coli* DH5α- pWBAD30- *sodB*-H28A | *E. coli* DH5α carrying plasmid construct pWBAD30-*sodB-*H28A mutant for complementation, Apr^R^, Kan^R^ | This study |
| *E. coli* DH5α carrying pWBAD30-*sodB*-D164A-H168A mutant | *E. coli* DH5α- pWBAD30- *sodB*-D164A-H168A | *E. coli* DH5α carrying plasmid construct pWBAD30-*sodB-*D164A-H168A mutant for complementation, Apr^R^, Kan^R^ | This study |
| *E. coli* DH5α carrying pWBAD30-*sodC* (native) with signal peptide | *E. coli* DH5α- pWBAD30-*sodC* | *E. coli* DH5α carrying plasmid construct pWBAD30-*sodC* (native) for complementation, Apr^R^, Kan^R^ | This study |
| *E. coli* DH5α carrying pWBAD30-*sodC*-H87A-H89A mutant with signal peptide | *E. coli* DH5α- pWBAD30- *sodC*-H87A-H89A | *E. coli* DH5α carrying plasmid construct pWBAD30-*sodC-*H87A-H89A mutant for complementation, Apr^R^, Kan^R^ | This study |
| *E. coli* DH5α carrying pWBAD30- *sodC*-H165A mutant with signal peptide | *E. coli* DH5α- pWBAD30- *sodC*-H165A | *E. coli* DH5α carrying plasmid construct pWBAD30-*sodC-H165A* mutant for complementation, Apr^R^, Kan^R^ | This study |
| *Acinetobacter baumannii* 5075 Δ*sodB*-pWBAD30 | Δ*sodB*- pWBAD30 | *sodB* k/o in *A. baumannii* 5075 with pWBAD30 (vector control), Apr^R^ | This study |
| *Acinetobacter baumannii* 5075 Δ*sodB*- pWBAD30*sodB* | Δ*sodB*- pWBAD30*sodB* | *sodB* k/o in *A. baumannii* 5075 with pWBAD30*sodB* (native *sodB* complementation), Apr^R^ | This study |
| *Acinetobacter baumannii* 5075 Δ*sodB*- pWBAD30*sodB-*H28A | Δ*sodB*- pWBAD30*sodB*-H28A | *sodB* k/o in *A. baumannii* 5075 with pWBAD30*sodB*-H28A for complementation, Apr^R^ | This study |
| *Acinetobacter baumannii* 5075 Δ*sodB*- pWBAD30*sodB-*D164A-H168A | Δ*sodB*- pWBAD30*sodB*-D164A-H168A | *sodB* k/o in *A. baumannii* 5075 with pWBAD30*sodB*-D164A-H168A for complementation, Apr^R^ | This study |
| *Acinetobacter baumannii* 5075 Δ*sodC*-pWBAD30 | Δ*sodC*- pWBAD30 | *sodC* k/o in *A. baumannii* 5075 with pWBAD30 (vector control), Apr^R^ | This study |
| *Acinetobacter baumannii* 5075 Δ*sodC*- pWBAD30*sodC* | Δ*sodB*- pWBAD30*sodC* | *sodC* k/o in *A. baumannii* 5075 with pWBAD30*sodC* (native *sodC* complementation), Apr^R^ | This study |
| *Acinetobacter baumannii* 5075 Δ*sodC*- pWBAD30*sodC*-H87A-H89A | Δ*sodC*- pWBAD30*sodC*-H87A-H89A | *sodC* k/o in *A. baumannii* 5075 with pWBAD30*sodC*-H87A-H89A for complementation, Apr^R^ | This study |
| *Acinetobacter baumannii* 5075 Δ*sodC*-pWBAD30*sodC-*H165A | Δ*sodC*- pWBAD30*sodC*-H165A | *sodC* k/o in *A. baumannii* 5075 with pWBAD30*sodC*-H165A for complementation, Apr^R^ | This study |

**Table S6.** List of plasmids used in this study.

| **Strains** | **Name used in this study** | **Description** | **Source** |
| --- | --- | --- | --- |
| pUC18 | N/A | Cloning vector, Amp^R^ | Purchased from Thermo Scientific,  USA |
| pET28a | N/A | Vector for expression of his-tagged recombinant  proteins in *E. coli*; Kan^R^ | Purchased from Novagen |
| pET28a-*sodB* (native) | pET28a-*sodB* | Plasmid for over expressing AB5075 SodB (native), Kan^R^ | This study |
| pET28a-*sodB-*H28A mutant | pET28a-*sodB-*H28A | Plasmid for over expressing pET28a-*sodB-*H28A mutant, single mutation at 28^th^ amino acid position (Histidine to Alanine) for over expressing AB5075 SodB-H28A mutant, Kan^R^ | This study |
| pET28a-*sodB-*D164A-H168A mutant | pET28a-*sodB-*D164A-H168A | Plasmid for over expressing pET28a-*sodB-*D164A-H168A mutant, double mutation at 164^th^ and 168^th^ amino acid positions (Aspartate to Alanine and Histidine to Alanine, respectively) for over expressing AB5075 SodB-D164A-H168A mutant, Kan^R^ | This study |
| pET28a-*sodc* (native) | pET28a-*sodc* | Plasmid for overexpressing AB5075 SodC (native), Kan^R^ | This study |
| pET28a-*sodC-*H87A-H89A mutant | pET28a-*sodC-* H87A-H89A | Plasmid construct pET28a-*sodC-*H87A-H89A mutant, double mutation at 87^th^ and 89^th^ amino acid positions (Histidine to Alanine) for over expressing AB5075 SodC-H87A-H89A mutant, Kan^R^ | This study |
| *E. coli* DH5α carrying pET28a-*sodC-*H165A mutant | *E. coli* DH5α- pET28a-*sodC-*H165A | Plasmid construct pET28a-*sodB-*H165A mutant, single mutation at 165^th^ amino acid position (Histidine to Alanine) AB5075 SodB- H165A mutant, Kan^R^ | This study |
| pMDIAI | N/A | Plasmid carrying apramycin (Apm^R^) resistance cassette  flanked by FRT sites | Purchased from Addgene |
| pWBAD30-Apr^R^ | pWBAD30 | Modified from pWBAD30-Kan^R^ harboring apramycin resistance cassette, arabinose pBAD promoter, Apr^R^ | This study |
| pWBAD30-*sodB* (native) | pWBAD30-*sodB* | Plasmid construct pWBAD30-*sodB* (native) for complementation, Apr^R^, Kan^R^ | This study |
| pWBAD30-*sodB*-H28A mutant | pWBAD30- *sodB*-H28A | Plasmid construct pWBAD30-*sodB-*H28A mutant for complementation, Apr^R^, Kan^R^ | This study |
| pWBAD30-*sodB*-D164A-H168A mutant | pWBAD30- *sodB*-D164A-H168A | Plasmid construct pWBAD30-*sodB-*D164A-H168A mutant for complementation, Apr^R^, Kan^R^ | This study |
| pWBAD30-*sodC* (native) with signal peptide | pWBAD30-*sodC* | Plasmid construct pWBAD30-*sodC* (native) for complementation, Apr^R^, Kan^R^ | This study |
| pWBAD30-*sodC*-H87A-H89A mutant with signal peptide | pWBAD30- *sodC*-H87A-H89A | Plasmid construct pWBAD30-*sodC-*H87A-H89A mutant for complementation, Apr^R^, Kan^R^ | This study |
| pWBAD30- *sodC*-H165A mutant with signal peptide | pWBAD30- *sodC*-H165A | Plasmid construct pWBAD30-*sodC*-H165A mutant for complementation, Apr^R^, Kan^R^ | This study |

**Table S7.** List of oligonucleotides used in this study.

| **Primer name** | **Sequence (5’ - 3’)** | **Description** | **Source** |
| --- | --- | --- | --- |
| *sodB*_*NheI*_FP | AACGGCTAGCATGACAACCATTACTTTACC | Forward primer for cloning native *sodB* into pET28a plasmid vector between NheI and XhoI sites | This study |
| *sodB_XhoI*_RP | AACCCTCGAGTTATTTCTCTACACCAGCTGG | Reverse primer for cloning native *sodB* into pET28a plasmid vector between NheI and XhoI sites | This study |
| *sodC_NheI*_FP | AACGGCTAGCTGTAGCACGGTAAGTAAGAC | Forward primer for cloning native *sodC* into pET28a plasmid vector between NheI and XhoI sites (without signal peptide) | This study |
| *sodC_XhoI*_RP | AACCCTCGAGTTATTTGATTACACCACATGCG | Reverse primer for cloning native *sodC* into pET28a plasmid vector between NheI and XhoI sites (without signal peptide) | This study |
| *sodB*_*EcoRI*_FP | CCGGAATTCATGACAACCATTACTTTACC | Forward primer for cloning native *sodB* into pWBAD30 between EcoRI and KpnI sites (for *in vivo* study) | This study |
| *sodB_KpnI*_RP | CGGGGTACCTTATTTCTCTACACCAGCTGG | Reverse primer for cloning native *sodB* into pWBAD30 between EcoRI and KpnI sites (for *in vivo* study) | This study |
| *sodB*_*H28A*_FP | GAATACGCTCACGATAAACACCACAATACCTATG | Forward primer for generating site directed mutagenesis in native *sodB* at 28^th^ amino acid position; mutagenesis was performed in both pET28a and pWBAD30 plasmid vectors | This study |
| *sodB_H28A*_RP | GTTTATCGTGAGCGTATTCTAAAGTTTCTTTACTGATATG | Reverse primer for generating site directed mutagenesis in native *sodB* at 28^th^ amino acid position; mutagenesis was performed in both pET28a and pWBAD30 plasmid vectors | This study |
| *sodB*_*D164A-H168A_*FP | CTATTGCTGTATGGGAAGCTGCTTACTACATCGATTTCC | Forward primer for generating site directed mutagenesis in native *sodB* at 164^th^ and 168^th^ amino acid positions (Aspartate to Alanine and Histidine to Alanine, respectively); mutagenesis was performed in both pET28a and pWBAD30 plasmid vectors | This study |
| *sodB*_*D164A-H168A_*RP | GTAAGCAGCTTCCCATACAGCAATAGTTAATACAGC | Reverse primer for generating site directed mutagenesis in native *sodB* at 164^th^ and 168^th^ amino acid positions (Aspartate to Alanine and Histidine to Alanine, respectively); mutagenesis was performed in both pET28a and pWBAD30 plasmid vectors | This study |
| *sodC*_*H87A-H89A*_FP | GGTTTCGCTATCGCTGAAAATCCATCTTGTGCCCCTGCTG | Forward primer for generating site directed mutagenesis in native *sodC* at 87^th^ and 89^th^ amino acid positions (Histidine to Alanine); mutagenesis was performed in both pET28a and pWBAD30 plasmid vectors | This study |
| *sodC*_*H87A-H89A*_RP | GGATTTTCAGCGATAGCGAAACCGCGAGTACCACTTGG | Reverse primer for generating site directed mutagenesis in native *sodC* at 87^th^ and 89^th^ amino acid positions (Histidine to Alanine); mutagenesis was performed in both pET28a and pWBAD30 plasmid vectors | This study |
| *sodC*_*H165A*_FP | GCTATCATGATCGCTGCTGGTGGTGATAACTATTCAG | Forward primer for generating site directed mutagenesis in native *sodC* at 165^th^ amino acid position (Histidine to Alanine); mutagenesis was performed in both pET28a and pWBAD30 plasmid vectors | This study |
| *sodC*_*H165A*_RP | CCAGCAGCGATCATGATAGCGCGACCTTGAATG | Reverse primer for generating site directed mutagenesis in native *sodC* at 165^th^ amino acid position (Histidine to Alanine); mutagenesis was performed in both pET28a and pWBAD30 plasmid vectors | This study |
| *sodC*_*EcoRI_*FP*-*pWBAD30 with signal peptide | GATTACGAATTCATGCCAGTATTTAATAAAATTGGTGCAGTATGTGCACTGACAGCTTTATTAACGGTCGGTTGTAGCAC | Forward primer for cloning native *sodC* start codon into pWBAD30 plasmid vector between EcoRI and KpnI sites (N-terminal 20 amino acid long signal peptide is present in cloned *sodC*) | This study |
| Apr_ClaI_FP_pWBAD30 | ATCAATCGATGTCGACCTGCAGTTC | Forward primer for cloning Apramycin resistance marker into pWBAD30 plasmid vector | This study |
| Apr_ClaI_RP_pWBAD30 | ATATCGATGTGTAGGCTGGAGCTGCTTC | Reverse primer for cloning Apramycin resistance marker into pWBAD30 plasmid vector | This study |
